# A Rotating Spiral Micromotor for Noninvasive Zygote Transfer

**DOI:** 10.1002/advs.202000843

**Published:** 2020-07-21

**Authors:** Lukas Schwarz, Dmitriy D. Karnaushenko, Franziska Hebenstreit, Ronald Naumann, Oliver G. Schmidt, Mariana Medina‐Sánchez

**Affiliations:** ^1^ Institute for Integrative Nanosciences Leibniz IFW Dresden Helmholtzstrasse 20 01069 Dresden Germany; ^2^ Transgenic Core Facility Max Planck Institute for Molecular Cell Biology and Genetics Pfotenhauerstrasse 108 01307 Dresden Germany; ^3^ Material Systems for Nanoelectronics Technische Universität Chemnitz Reichenhainer Strasse 70 09126 Chemnitz Germany; ^4^ Nanophysics Technische Universität Dresden Nöthnitzer Strasse 61 01187 Dresden Germany

**Keywords:** assisted reproduction, micromotors, micropropellers, rotating magnetic fields, spirals, zygote intrafallopian transfer

## Abstract

Embryo transfer (ET) is a decisive step in the in vitro fertilization process. In most cases, the embryo is transferred to the uterus after several days of in vitro culture. Although studies have identified the beneficial effects of ET on proper embryo development in the earlier stages, this strategy is compromised by the necessity to transfer early embryos (zygotes) back to the fallopian tube instead of the uterus, which requires a more invasive, laparoscopic procedure, termed zygote intrafallopian transfer (ZIFT). Magnetic micromotors offer the possibility to mitigate such surgical interventions, as they have the potential to transport and deliver cellular cargo such as zygotes through the uterus and fallopian tube noninvasively, actuated by an externally applied rotating magnetic field. This study presents the capture, transport, and release of bovine and murine zygotes using two types of magnetic micropropellers, helix and spiral. Although helices represent an established micromotor architecture, spirals surpass them in terms of motion performance and with their ability to reliably capture and secure the cargo during both motion and transfer between different environments. Herein, this is demonstrated with murine oocytes/zygotes as the cargo; this is the first step toward the application of noninvasive, magnetic micromotor‐assisted ZIFT.

## Graphical abstract

Spiral micropropellers that capture, transport, and release oocytes and zygotes, actuated solely by a rotating magnetic field, and are transferred between different environments without losing the cargo, are presented. The propulsion and transport performance of the innovative propellers and their advantages over established helical micromotors are demonstrated regarding applicability in a noninvasive alternative concept to laparoscopy, zygote transfer by micromotors.

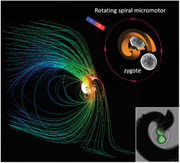

## 1 Introduction

Micromotors, untethered devices that move and operate at a microscale, are especially relevant for biomedical applications, potentially enabling localized and controllable material‐to‐cell interactions for therapeutic,^[^ ^1^, ^2^, ^3^ ^]^ diagnostic,^[^ ^4^, ^5^ ^]^ or microsurgical^[^ ^6^, ^7^ ^]^ purposes in vivo, that is, operating inside the patient's body.^[^ ^8^ ^]^ Micromotors can be actuated and controlled with chemicals in their surroundings^[^ ^9^, ^10^, ^11^ ^]^ or physically by external power sources such as light,^[^ ^12^ ^]^ ultrasound,^[^ ^13^ ^]^ or electric^[^ ^14^ ^]^ or magnetic fields.^[^ ^15^, ^16^, ^17^ ^]^ Motile microorganisms such as flagellated bacteria^[^ ^18^, ^19^ ^]^ or sperm cells^[^ ^20^, ^21^ ^]^ can be integrated to form a biohybrid micromotor that is driven by the on‐board power supply of the microorganism. Sperm cells can not only provide propulsive power, for example, when applied as carriers for targeted drug delivery,^[^ ^1^, ^22^ ^]^ but can also be supported by micromotors for their original purpose, that is, fertilization.^[^ ^20^, ^23^ ^]^ The concept of micromotor‐assisted fertilization was first demonstrated with magnetic microtubes coupled to individual sperm cells to allow the magnetic guidance of the sperm.^[^ ^20^ ^]^ Later, magnetically propelled microhelices were developed for coupling to immotile sperm cells and transporting them to the oocyte.^[^ ^23^ ^]^ Both sperm‐hybrid micromotor approaches were designed to remedy sperm deficiencies, and thus counter male infertility. Unlike conventional in vitro fertilization (IVF) and intracytoplasmic sperm injection (ICSI), micromotor‐assisted fertilization concepts offer the opportunity to be applied in vivo, that is, micromotors can support the movement of the sperm cells through the uterus and fallopian tubes. This implies that it would not be necessary to explant oocytes and culture them in the laboratory, and fertilization can occur naturally. The same advantage applies to the application of magnetic micromotors to the assisted reproductive technology that we propose in this work, which addresses a later stage of the fertilization process, that is, embryo development. Early embryo development is the phase after fertilization, that is, fusion of the sperm and oocyte, where the fertilized oocyte, termed zygote, completes its journey through the fallopian tube and undergoes several cell divisions before it “hatches” from its glycoprotein shell, the zona pellucida, and becomes implanted in the uterine wall.^[^ ^24^, ^25^ ^]^ In the case of IVF (and ICSI), after fertilization, zygotes are cultured for several days in the laboratory before they are reimplanted into the uterus.^[^ ^26^, ^27^, ^28^ ^]^ Considering the high success rate of IVF/ICSI, which is more than 90%, and the comparatively low rate of successful pregnancies after embryo transfer (ET), which is marginally more than 30%,^[^ ^26^, ^27^, ^28^ ^]^ the period of in vitro zygote culture and embryo development before reimplantation and the ET procedure would appear to be critical steps that currently compromise the entire IVF process. Zygote intrafallopian transfer (ZIFT) is a concept that has been devised to address this issue. If zygotes are transferred shortly after IVF/ICSI, they can undergo early embryo development in their natural environment and benefit from an optimal synchronization between embryonic and endometrial development^[^ ^29^, ^30^ ^]^; however, to achieve this, they must be transferred back to the fallopian tube, not the uterus. This procedure is considerably more complex than uterine ET, as it requires laparoscopic surgery through incisions into the pelvis to access the fallopian tube with a cannula (and camera).^[^ ^31^ ^]^ Having been applied regularly in the clinic since the 1980s, ZIFT has proven its advantage over uterine ET in cases of non‐tubal factor infertility, especially after repeated implantation failure (RIF)^[^ ^30^ ^]^; however, a general superiority of the method could not be verified statistically at this point.^[^ ^32^, ^33^, ^34^ ^]^ Presumably, for two reasons, the advantage of natural embryo development in the fallopian tube after ZIFT does not benefit all cases. ^[^ ^32^, ^33^ ^]^ First, transferring zygotes shortly after IVF/ICSI limits the means to assess and confirm the embryo quality before transfer, and one loses the opportunity to select the best of several embryos after several days of in vitro culture.^[^ ^32^, ^33^, ^35^ ^]^ This is especially critical as the practice of elective single embryo transfer (eSET), that is, transferring only one single zygote/embryo to avoid multiple pregnancies, is becoming increasingly important.^[^ ^26^, ^27^, ^28^ ^]^ Second, the laparoscopic surgery procedure entails risks of inflammation, infection, and intraluminal pathologies and imposes stress on the patient's body, which can compromise the entire pregnancy in its early stage.^[^ ^30^, ^32^, ^33^ ^]^ This is an aspect where the novel concept of employing micromotors for assisted reproductive technology can provide a substantial improvement, considering the untethered and therefore noninvasive, externally controlled operation of micromotors for in vivo applications. In this work, we demonstrate the possibility of capturing, transporting, and releasing zygotes with magnetically actuated micromotors as a proof‐of‐concept for micromotor‐assisted ZIFT. We present the novel design of a spiral‐shaped micromotor and demonstrate its advantages over the established helical micromotor design^[^ ^16^, ^17^ ^]^ considering the application of cell transport and delivery. Specifically, we demonstrate efficient propulsion and cargo delivery in high‐viscosity fluids and confined channels, highlighting the spiral's unique ability to secure the cellular cargo during magnetically actuated transport as well as during transfer between the different environments by pipetting. Furthermore, we provide a detailed analysis of the propulsion mechanism and performance of the spiral‐shaped micropropeller compared to the helix, based on experimental findings and fluid dynamics simulations. Regarding alternative strategies for cargo transport by micromotors,^[^ ^36^, ^37^, ^38^, ^39^, ^40^ ^]^ we demonstrate the simplicity, controllability, and reliability of the proposed approach, dependent solely on an external, homogeneous, rotating magnetic field, and the geometric architecture of the micromotor. This is achieved with no other physical or chemical triggers for the cargo capture and release, and no complex arrangement of the different necessary micromotor components that could compromise the feasibility or biocompatibility. Several works have demonstrated cargo transport where particles or cells are not simply pushed by or irreversibly adhered to a micromotor. However, resilient and reversible cargo loading, for example, with microscopic grippers^[^ ^36^, ^37^ ^]^ or case‐and‐lid mechanisms,^[^ ^40^ ^]^ have been demonstrated to be dependent on triggers such as heat,^[^ ^36^ ^]^ pH,^[^ ^37^ ^]^ light,^[^ ^38^ ^]^ an electric field,^[^ ^39^ ^]^ or multiple steps of magnetically controlled assembly.^[^ ^40^ ^]^ Their in vivo application is difficult (and risky), particularly when referring to the task of transporting fertilized oocytes, as these triggers could compromise their viability and induce oxidative stress. Thus, in the present work, simple magnetic actuation by a homogeneous, rotating field of up to 20 mT, chosen for its biocompatibility, is demonstrated to be sufficient for resilient and reversible cargo delivery, employing an innovative micromotor architecture for the intended in vivo application of micromotor‐assisted ZIFT. Although different applications of cell capture and transport can be envisioned with the micromotors presented in this work, for example, as motile scaffolds for tissue engineering^[^ ^40^ ^]^ or local stem cell delivery,^[^ ^41^ ^]^ our demonstrations focus on the manipulation of single zygotes for noninvasive ZIFT as a form of eSET.

## 2 Results and Discussion

### 2.1 Concept and Design

**Figure 1:**
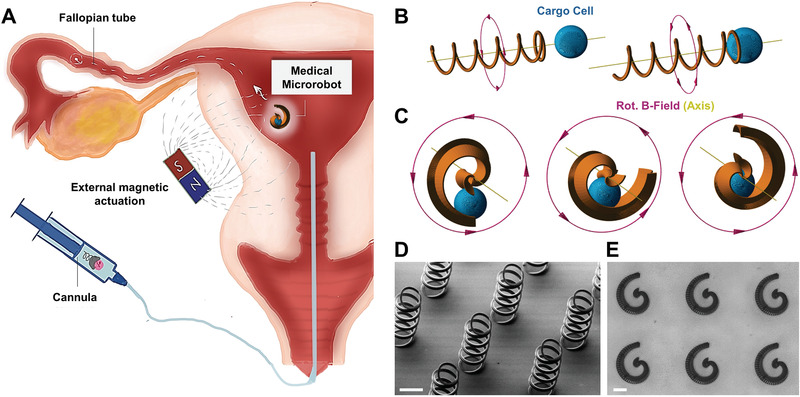
Micromotor concept and design: A) Proposed micromotor‐assisted ZIFT procedure; B) cargo pushing with helix; C) cargo capture with spiral; D) fabricated helices with head rings and five windings, 100 µm diameter, and 50 µm pitch length (scale bar: 100 µm); E) fabricated spirals with 390 µm footprint length and 170 µm opening diameter (scale bar: 100 µm); for dimensions and details of design features please refer to Figure S1, Supporting Information.

**Figure 2:**
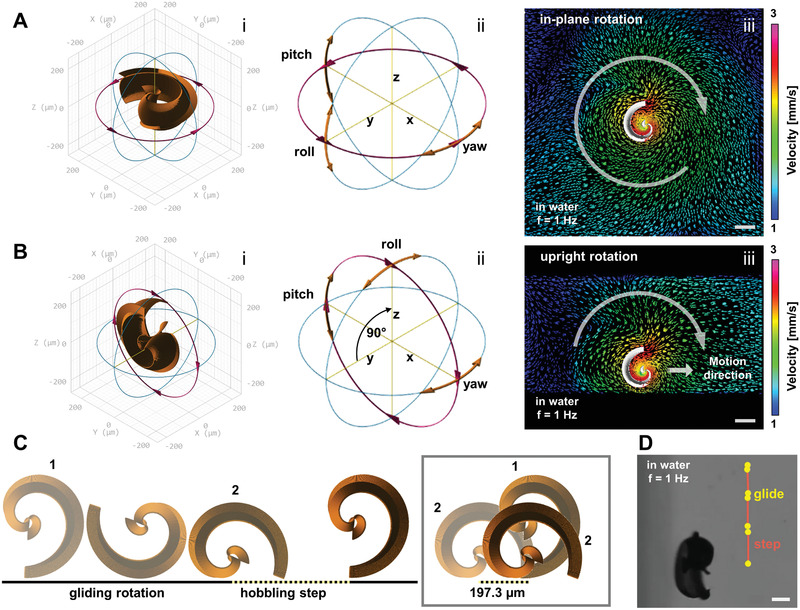
Propulsion principle of magnetic spirals: A) In‐plane rotation on surface (i), actuated by rotating magnetic field whose spatial orientation can be tilted by “roll,” “pitch,” and “yaw” parameters (ii); B) upright rotation on surface (i), “roll” set to 90°, “yaw” used for steering the instating forward propulsion (ii), modeling images of fluid flow velocities around rotating spiral by ANSYS 17.2 Academic software in (A,iii) and (B,iii) indicate obtained directionality (marked with white arrows in both cases and additional straight arrow in upright case); C) gliding and stepping motion of spiral close to substrate surface (step length 197.3 µm); D) inverted video microscopy image of spiral moving forward on surface in water, rotating with 1 Hz, tracked with marks after every 15 frames (video framerate 30 fps), illustrating uneven hobbling motion, all scale bars: 100 µm.

The concept of micromotor‐assisted ZIFT is illustrated in **Figure** **1**A. Avoiding laparoscopy, the internal space of the fallopian tube cannot be reached with a cannula directly; only the uterine cavity can be accessed. The transfer must be accomplished by a micromotor that can capture, transport, and release an individual zygote in a resilient and reversible manner under external control. Two different micromotor architectures, both actuated by a rotating magnetic field, are investigated in this work to complete this task. One is based on a helical corkscrew propeller, a well‐established magnetic micromotor architecture.^[^ ^16^, ^17^, ^23^, ^40^ ^]^ The other is an innovative design of a spiral‐shaped micropropeller. The helical design is suitable to simply push cellular cargo while propelling forward (Figure 1B), whereas the spiral shape can enclose and protect cargo by rotating around it to fix it in the spiral's center (Figure 1C). Both mechanisms are reversible, as cargo can be released upon inversion of the micromotor's rotation direction, which propels the helix away from the cargo, and rotates the spiral's body away to expose the enclosed cargo particle or cell. Fabricated by 3D laser lithography (based on two‐photon absorption), both micromotors can be freely scaled in size to fit polystyrene (PS) particles (100 µm diameter), bovine oocytes/zygotes (≈120–150 µm), or murine oocytes/zygotes (≈50–80 µm). For helices, the geometrical requirement to transport such spherical cargo is simply to have a diameter that is marginally smaller, for example a head ring of 100 µm diameter to capture bovine zygotes, as shown in Figure 1D. Spirals, however, must be sufficiently large to completely enclose the respective cargo sphere. Moreover, the tubular diameter, that is, the diameter of the spiral's opening, must be sufficiently small to guide the cargo along the half‐tubular spiral wall toward the center, not allowing it to escape through the open sides of the spiral. For example, to capture a bovine zygote, an opening diameter of 170 µm was suitable, as indicated in Figure 1E. Smaller spirals to capture murine zygotes were also fabricated and scaled down with a tubular opening diameter of 150, 130, and 110 µm. Based on experimental observations, a helical fin was added along the backbone of the spiral geometry, as displayed in Figure 1C,E and described in Figure S1, Supporting Information. The fin's influence on the propulsion performance of the spiral is discussed below. The mechanism of rotating chiral structures that propel forward in a low Reynolds number environment, actuated by a rotating magnetic field, is well known, especially for helical micropropellers.^[^ ^42^, ^43^ ^]^ The innovative spiral‐shaped micropropellers presented in this work are actuated in the same manner, yet behave differently. Helices rotate around their long axis in their stable mode of propulsion and propel forward along that axis because of drag anisotropy, provided they are sufficiently magnetized to follow the rotation of the externally applied magnetic field, depending on the rotation frequency, as indicated in Figure 1B. Spirals rotate preferably around their short axis, as indicated in Figure 1C, over a wide range of actuation frequencies. While lying flat on the substrate, as in Figure 1E and sketched in **Figure** **2**A‐i, no significant forward propulsion was observed upon (in‐plane) rotation (Video S1, Supporting Information). When the rotation axis of the external field was tilted to force the spiral to “stand up” (Figure 2B‐i), it began to roll, similar to a wheel, on the substrate floor (Video S1, Supporting Information). Helices can also perform such rolling actions when swimming close to the substrate floor, noticeable as an off‐axis side drift perpendicular to their corkscrew propulsion direction,^[^ ^44^ ^]^ which exacerbates directional control in this case. Next, the rolling motion of the spirals is analyzed in detail and the advantages over helical corkscrew propulsion is discussed. Experimentally, both helices and spirals are propelled and steered in a liquid medium by manually controlling the external magnetic field's rotation axis with a 3D mouse to which both micromotors respond without noticeable time lag. The rotating magnetic field can be tilted along all spatial axes by setting the parameters “roll,” “pitch,” and “yaw,” as sketched in Figure 2A‐ii,B‐ii (please refer to the Experimental Section and Figure S1, Supporting Information, for details on the experimental setup). Helices exhibit the previously described well‐known corkscrew propulsion behavior, whereas upright spirals (roll = 90°, as indicated in Figure 2B‐ii) appear to roll on the respective substrate surface in water, similar to rolling microcoils that have been studied elsewhere.^[^ ^45^ ^]^ However, this rolling motion is not characterized by a direct translation of the spiral circumference length to the distance traveled; rather, it can be described as a two‐stage process of gliding and stepping. From the initial position depicted on the left of Figure 2C (position “1”), an upright standing spiral that rotates in the clockwise direction glides along the substrate surface while the fin along its rounded backbone prevents the majority of the microstructure's surface from touching the substrate. In this phase, no significant forward propulsion is observed, similar to the case of a spiral rotating lying flat on the surface as indicated in Figure 2A‐iii, where the modeling image (by ANSYS 17.2 Academic) indicates no forward directionality of flow around the rotating spiral (see also Video S2, Supporting Information, with different color scaling). The second phase of the upright rolling begins when the opening of the spiral draws near the surface, that is, when the endpoint of the fin is reached and the smooth gliding is interrupted by the spiral's tip connecting with the ground (position 2 in Figure 2C). The spiral hobbles and flips over to reach the initial position and begins gliding again (marked as “hobbling step” in Figure 2C). This stepping motion constitutes the main component of the forward propulsion, with a step length of 197.3 µm according to the spiral geometry (with an opening diameter of 170 µm). Experimentally, it has been observed that the spiral does not move forward in the first half of its moving cycle, as indicated in Figure 2D and Video S3, Supporting Information, which depicts a tracking experiment at 1 Hz actuation frequency in water, recorded at 30 fps. Each track mark was made after 15 frames, that is, half a rotation. Clearly, the track marks are spaced unevenly according to the hobbling motion of the spiral while moving from the top to bottom of the image (please note that all spirals were recorded inversely, that is, from below the substrate in this work). According to the directionality of the flow that is imposed by the spiral opening, which disturbs the smooth gliding in close vicinity to the substrate floor (Figure 2B‐ii; see also Video S2, Supporting Information), the two phases of spiral motion can actually counteract each other, that is, a spiral can initially glide backwards while rotating smoothly and then step forward over its opening gap. This has been observed experimentally in a high‐viscosity medium, making it difficult to accurately model the propulsion speed quantitatively, which is discussed below. Interestingly, the chirality of the fin along the backbone of a spiral, which renders the micromotor's rotational motion nonreciprocal, does not appear to be a necessary requirement for forward propulsion, considering the fact that the spiral's propulsion is surface‐mediated and functions similar to the simpler rotating/tumbling micropropellers reported in earlier works.^[^ ^46^, ^47^ ^]^ Nonetheless, spirals without fins did not exhibit efficient forward propulsion in the corresponding experiments. Presumably, a more stable rotation behavior and a minimization of direct contact with the substrate surface (that is, friction) are advantages of the fin structure that benefit spiral propulsion. Additional magnetization of the fin could also be advantageous.

### 2.2 Propulsion Performance of Helices and Spirals

**Figure 3:**
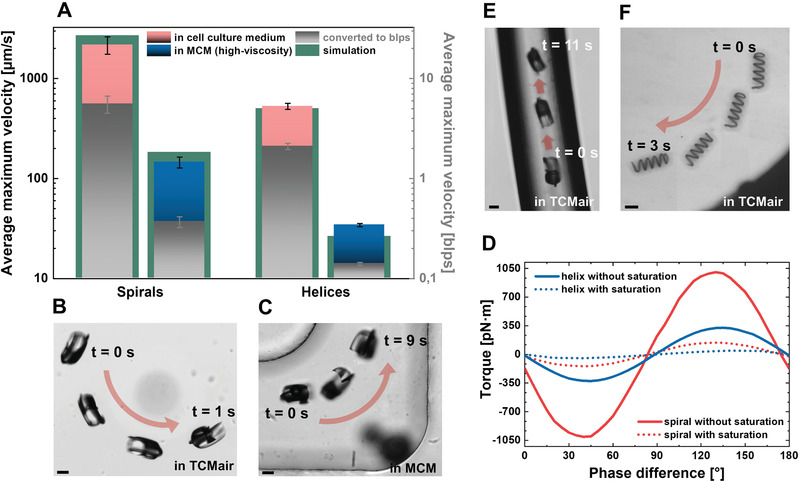
Performance of helices and spirals: A) Average velocities of spirals (*n* = 9) and helices (*n* = 14) in different media and converted to blps (gray axis and columns), error bars indicate standard deviations between different individual micromotors (experimental data), please note logarithmic scaling; B) spiral propulsion in inlet region of PDMS channel in cell culture medium TCMair; C) spiral propulsion in PDMS channel in high‐viscosity medium MCM (0.6 w/v methyl cellulose, viscosity ≈25 mPa s); D) modeling results (ANSYS simulations) of maximum torque generated by helix and spiral‐shaped micromotors in homogeneous magnetic field of 20 mT, depending on their saturation magnetization and resulting phase difference between rotating magnetic field and respective microstructure, for details on distribution of ferromagnetic material on respective microstructure please refer to Figure S1, Supporting Information; E) spiral propulsion in narrow PTFE channel (Ø = 500 µm) in TCMair; F) helix propulsion in parafilm channel in TCMair, all scale bars: 100 µm.

The experimental results of the propulsion experiments of the spirals and helices in different environments are presented in **Figure** **3** and Video S4, Supporting Information. The spirals were actuated in water‐based bovine and murine oocyte/zygote cell culture medium (TCMair and M2, respectively; see Experimental Section) and in cell culture medium with 0.6 w/v methyl cellulose (MCM). MCM was composed to mimic the high viscosity of real oviduct fluid (OVF), which was isolated from bovine oviducts from different cows in only extremely small quantities. Shear rheometry revealed dynamic viscosities of ≈20–25 mPa s for the MCM and OVF, which is ≈20‐fold that of water at 20 °C. The helices were actuated in TCMair, MCM, and OVF. A comparison of the average maximum velocities of the spirals and helices in cell culture media and high‐viscosity MCM is depicted in Figure 3A. The average maximum velocities were tracked while the micromotors were swimming through microfluidic channels made from poly(dimethylsiloxane) (PDMS) or parafilm on glass, and the helix velocities were preferably tracked when there was no side drift, which could not always be avoided. Spirals exhibited no significant drift and typically followed the intended direction (i.e., rotating magnetic field alignment) accurately. As can be observed in Figure 3A, spirals typically achieve greater velocities than helices in a water‐based cell culture medium (pink columns). The depicted maximum velocity of the spirals (≈2000 µm s^−1^) was achieved with an actuation frequency of 25 Hz. In the majority of the experiments, the spirals were not operated at such high frequencies when swimming through the channels, as their rapid pace would make it difficult to follow their movements under a microscope, and they may quickly move out of the field of view (at 10× magnification). Such high velocities were measured with a fixed field of view, allowing a spiral to move in and out of view on circular tracks in the inlet region of a PDMS channel, as depicted in Figure 3B (see also Video S4, Supporting Information). Moderate velocities of ≈800 µm s^−1^ were typically achieved when propelling through a PDMS channel in the range of 4–7 Hz actuation frequency in TCMair and M2. These approximately match the average maximum velocities of the helices in the same medium in terms of body lengths per second (blps), indicated by the gray columns, with a value of ≈2 blps for both micromotors. However, helices required actuation at 50 Hz to achieve the depicted maximum velocity of ≈500 µm s^−1^ (please note the logarithmic scaling in Figure 3A). As reported in numerous studies on helical micropropellers,^[^ ^23^, ^40^, ^44^ ^]^ the velocity of a rotating helix in a low Reynolds number environment increases linearly with the actuation frequency and then drops abruptly at a certain frequency when the micromotor can no longer follow the rotation of the applied magnetic field owing to fluid drag,^[^ ^48^, ^49^ ^]^ depending on the magnetic field strength, micromotor magnetization, and geometry. Such behavior is not observed with the spiral‐shaped micromotors. The propulsion velocity of a spiral also increases linearly with the actuation frequency in a certain range (up to 10 Hz) and then saturates at higher frequencies. This behavior was also reported in other studies of rolling magnetic micropropellers^[^ ^50^, ^51^ ^]^ and is documented in more detail in Figure S3, Supporting Information. Although spirals, similar to helices, cannot follow the externally imposed rotation frequency at a certain point, this does not appear to prevent them from rotating with a reduced (saturated) frequency. However, it must be noted that, on occasion, spirals can switch to a tumbling mode, that is, begin to rotate around a different axis of their shape, with a considerably reduced forward propulsion. Tumbling presumably occurs owing to a local minimum of counter drag from the fluid at a given rotation regarding a certain micromotor orientation that is not favorable for propulsion. Depending on the phase difference between the external magnetic field rotation and the micromotor's rotation, there could be a bi‐stable state of favorable orientations (and rotation axes) of the spiral, as it performs skipping and ratchet‐like behavior when it cannot accurately follow the magnetic field's rotation.^[^ ^49^ ^]^ Such tumbling modes occurred at actuation frequencies greater than ≈20 Hz but could not be recognized to specific frequencies, as they appeared to be initialized by marginal deviations from the upright rolling position of the spiral relative to the substrate. These deviations occur especially when propelling around corners, as the in‐plane orientation (“yaw” for steering) and the tilt angle relative to the substrate (“roll,” ideally 90° as in Figure 2B) are frequently altered simultaneously with the manual control (3D mouse) of the magnetic actuation setup. Tumbling modes also depend on micromotor magnetization, which has been demonstrated and studied elsewhere with rolling microcoils.^[^ ^45^ ^]^ Quick saturation of the spiral propulsion was observed in the MCM at 2 Hz because of the medium's high viscosity (≈20–25 mPa s, ≈20× that of water at 20 °C). A significantly reduced average maximum velocity of spirals in MCM is indicated in Figure 3C and documented in Figure 3A, as well as for helices in the same medium (blue columns). Comparing helices in cell culture medium and MCM, the velocity difference was as striking as with spiral micromotors, with a decrease of more than 93% in both cases. As mentioned above, helices were also operated in OVF, which was available in only extremely small quantities. Significantly greater velocities were reached in OVF than in MCM (≈120 compared to 35 µm s^−1^, data not provided), which indicates that the hydrodynamic properties of OVF could not be accurately mimicked by MCM (i.e., by adding 0.6 w/v methyl cellulose to cell culture medium), as the performance of the helices in OVF proved to be significantly superior to that in MCM. This discrepancy can be explained by the fact that the rheological properties of OVF are heavily influenced by solid parts such as cells and cell debris that are dispersed in the fluid. The complex molecular and microscopic architecture of the fluid can evoke a different viscoelastic response, especially to the shear stress introduced by the rotating micromotor. Further investigations must be conducted to accurately reproduce this complex fluid and its hydrodynamic properties. From this study, it can be concluded that both spirals and helices can propel in MCM that, by comparison with the data on helices in OVF, appears to be an even more restrictive medium for micromotor propulsion than OVF. For both micromotors, helix and spiral, the significant velocity decrease in high‐viscosity media is mainly a consequence of their inability to follow the high rotation frequencies of the externally applied magnetic field. Regarding the corkscrew propulsion of helices, a higher medium viscosity theoretically leads to a higher fluid drag and torques, which should lead to a higher propulsion velocity, given that the helix's motion is based on drag anisotropy.^[^ ^42^, ^43^ ^]^ However, the microstructure must be able to follow the magnetic field to rotate, which is a matter of magnetization.^[^ ^45^, ^48^, ^49^ ^]^ Figure 3D displays the modeling results (by ANSYS 17.2 Academic software) of the maximum available torque for the helices and spirals as a function of the phase difference of the externally applied rotating magnetic field and the respective micromotor following that rotation, which depends on the microstructure's presumed saturation magnetization. An estimation of the distribution of the ferromagnetic material on the respective microstructures is provided in Figure S1, Supporting Information. The two graphs reflect the basic experimental finding that spirals yield higher torques (and therefore velocities) than helices. However, it should be noted that the spirals’ propulsion is not only defined by the torque and drag anisotropy that lead to a gliding motion similar to the helical corkscrew propulsion^[^ ^52^ ^]^ but also relies on the stepping motion described earlier (Figure 2C,D), which is mediated by direct contact with a substrate surface. Experimentally, it was observed that spirals can follow the magnetically imposed rotation of an external magnetic field of 20 mT accurately up to ≈20 Hz in a water‐based medium, yet only up to ≈1.5 Hz in MCM, based on a frame‐by‐frame examination of videos recorded at 30 fps. For helices, ≈40 and 2 Hz could be reproduced accurately. Although hydrodynamic simulations (details in the Experimental Section) provided reasonably accurate estimations of the maximum velocities at these frequencies for both microstructures (displayed in green in Figure 3A), they could not accurately predict the substantial limitation of achievable rotation frequencies in a high‐viscosity medium owing to the viscous damping effect. It should be noted that the model for the simulation of spiral propulsion was only valid for the hydrodynamic gliding component of its motion and generated velocities of opposite sign compared to the “hobbling step” motion that was identified as the main component of the forward propulsion (see Figure 2C,D). Such backward motion was observed experimentally during the first half of each spiral rotation cycle at low frequencies in MCM, and has also been reported in early works on simpler rotating/tumbling micropropellers.^[^ ^46^, ^47^ ^]^ Moreover, it was observed that when spirals propelled in the bulk medium, not in contact with the substrate floor, their swimming velocity switched sign and decreased significantly (Video S5, Supporting Information). Consequently, the simulated spiral velocities in Figure 3A were calculated as the difference of the modeled velocity subtracted from the product of the step length (197.3 µm, Figure 2C) and the respective maximum rotation frequency. Experiments were conducted to illustrate the importance of the stepping component of spiral propulsion on a surface; the externally applied direction of rotation was quickly switched while the frequency was held constant. Interestingly, the spirals always flipped back to the “correct” orientation to reestablish the stepping motion in a water‐based medium, whereas in MCM, they did not flip, and thus could not propagate efficiently. This was because the spiral opening could no longer act as a hinge point for stepping when the spiral rotation was inverted, and therefore only smooth, yet ineffective gliding was possible (Video S6, Supporting Information). The aforementioned viscous damping combined with the counter flow induced by spiral propulsion also impedes forward propulsion, especially in spatially confined environments, even in a water‐based cell culture medium; for example, when rolling through a narrow poly(tetrafluoroethylene) (PTFE) tubing (500 µm diameter, Figure 3E). It was verified experimentally that the rolling velocity of a spiral decreases linearly with spatial confinement, that is, the diameter of the channel, as indicated in Figure S3, Supporting Information. This was observed when allowing spirals to propel through a trimmed 10 µL pipette tip with an inner diameter of 500–1500 µm (see Experimental Section) with a constant actuation frequency, from the wide to the narrow tip end and back. In PTFE tubing (as in Figure 3E; Video S4, Supporting Information), the average maximum velocities of ≈85 µm s^−1^ in TCMair and ≈20 µm s^−1^ in MCM were measured because of the greater hydrodynamic resistance and viscous dissipation closer to the walls^[^ ^53^ ^]^ and the presumably enhanced effect of counter‐directional gliding propulsion. Apart from swimming in parafilm channels in TCMair (as in Figure 3F), MCM, and OVF, one helical micropropeller was also actuated successfully in MCM in a PTFE tubing and achieved an average maximum velocity of ≈25 µm s^−1^ at an actuation frequency of only 1.5 Hz. Apparently, helices suffer less from the confinement (500 µm tubing diameter) compared to spirals, as their long axis remains parallel to the channel, whereas spirals require space to rotate their full footprint size (390 µm, as indicated in Figure 1E). High medium viscosity and spatial confinement are two major constraints that must be considered when aiming for the in vivo application of biomedical micromotors. These two aspects have been addressed with the presented experiments, revealing the potential of both types of micromotors. However, it must be noted that the interior of a live oviduct is not only highly viscous and confining but also soft, convoluted, and ciliated.^[^ ^54^ ^]^ Moreover, there is a flow of oviduct fluid toward the uterine cavity. These conditions will be addressed and studied in detail in future studies. Although the quantitative propulsion performances of the helix and spiral under the investigated conditions do not appear to be fundamentally different from each other, the fact that helices are swimming freely in bulk fluid, whereas the propulsion of spirals is surface‐mediated, can be of importance regarding the aforementioned conditions in a live oviduct. It is possible that helical micromotors cannot swim freely, as contact with the epithelium could presumably be unavoidable in the described case. From the experimental observations, the stepping motion of the spirals allows them to propel along lateral and curved walls and to traverse cell debris. Furthermore, close proximity to the epithelial walls is expected to be advantageous when moving against fluid flow. Consequently, controllable surface‐mediated propulsion could be preferable to helical corkscrew propulsion in a live oviduct. Primarily, spiral‐shaped micromotors have a significant advantage over helices in the specific application of micromotor‐assisted ZIFT, that is, the transport of fertilized oocytes, which is demonstrated next.

### 2.3 Cell Transport and Delivery

**Figure 4:**
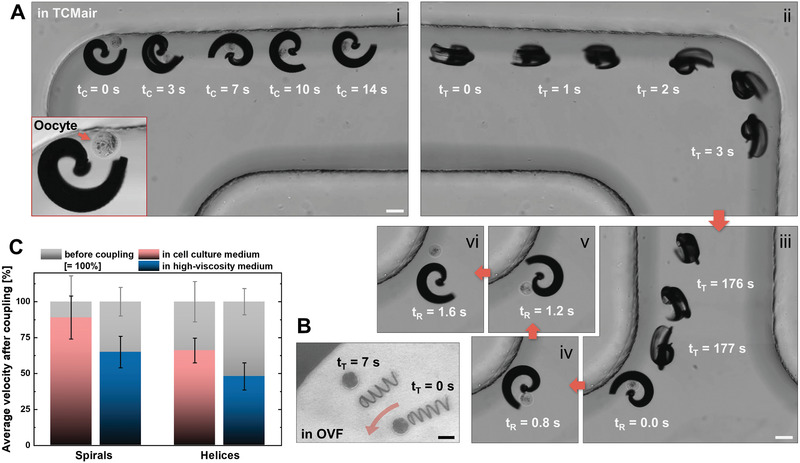
Cargo transport with helices and spirals: A) Capture (i), transport (ii,iii), and release (iv–vi) of murine zygote by spiral; B) failed transport of bovine zygote by helix, all scale bars: 100 µm; C) average velocities of spirals (*n* = 4) and helices (*n* = 11) after cargo coupling in low‐ and high‐viscosity media, normalized to respective cases (same medium, same individual micromotor) before coupling, error bars indicate standard deviations from velocity difference for different individual micromotors.

**Figure 5:**
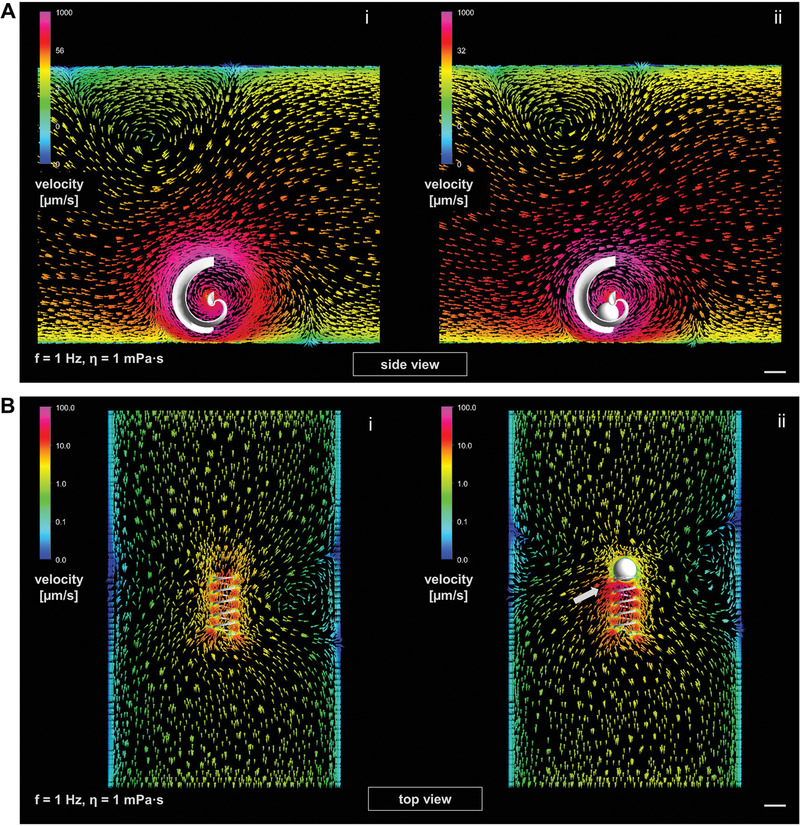
Fluid dynamics simulations (ANSYS 17.2 Academic software) of loaded and unloaded micromotors in water‐based medium (*η* ≈1 mPa s) at 1 Hz rotation after five complete rotations, logarithmic color scales of flow velocities around micromotors (different for spiral and helix) indicate fluid velocity: A) spiral without (i) and with (ii) spherical cargo (side view); B) helix without (i) and with (ii) spherical cargo (top view), white arrow marks a significant flow disturbance; all scale bars are set at 100 µm; please refer to Figure S4A,B, Supporting Information for results in high viscosity (1 mPa s).

A spiral‐shaped micromotor can enclose cargo by rotation, as indicated in **Figure** **4**A‐i with a murine oocyte (see also Video S7, Supporting Information). To capture the cell, the micromotor is required to move toward it during rotation. Therefore, it was not sufficient to rotate while lying flat on the substrate plane, as this would lead to virtually no forward propulsion. Consequently, the rotating magnetic field was marginally tilted by 10–15°, which was sufficient to propel the spiral toward the oocyte while remaining sufficiently flat to allow the cargo capture mechanism to be clearly visible by microscopy. Figure 4A‐ii,iii display the transport of the captured oocyte through the PDMS channel (Figure 4A‐ii is a mirror image of Figure 4A‐i at a later time (see also Video S7, Supporting Information). Ultimately, the oocyte is released by counter‐rotation at the other end of the channel, which is depicted in a series of still frame images (Figure 4A‐iv–vi), where the spiral was rotated in the substrate plane, lying flat on the glass substrate (see also Video S7, Supporting Information). Note that the spiral was scaled down to a tubular diameter of 130 µm instead of 170 µm to accommodate murine instead of bovine oocytes and zygotes. Several PS particles, bovine and murine oocytes, and zygotes were transported through PDMS channels by different spirals, as in the depicted example, moving back and forth through the channel over a total distance of several centimeters in several minutes in each case. Similar experiments were conducted with helices and bovine oocytes/zygotes; however, these cases suffered from a distinctive problem: helices could not always hold the cargo cell in place during the transport, that is, the cell was frequently lost along the micromotor's track, especially when swimming around corners, as depicted in Figure 4B, which displays a helix pushing a bovine zygote through a parafilm channel in OVF. Nonetheless, successful transport through the channels was possible in several cases because of the formation of a weak hydrodynamic vortex that pulled the captured cell toward the propelling helix, at least as long as the helix was rotating stably, which is discussed below (Video S8, Supporting Information), and by recapturing the lost cargo if necessary. Figure 4C displays the decrease in micromotor velocities after cargo capture and coupling, relative to the respective micromotor velocity before coupling. Because of the different cargo cells and particles, and owing to the fact that the micromotors were not always operated at their maximum possible velocity, all velocities after cargo coupling were normalized to the velocity before coupling of the same individual micromotor. Then, all the obtained velocity differences were averaged regarding the two types of micromotors, helix and spiral, and the two types of liquid medium, high (MCM, OVF) and low (TCMair, M2) viscosity, as can be observed in Figure 4C. Clearly, both micromotors propelled more slowly when loaded with cargo. This effect was less pronounced for spirals (≈90% of the respective initial velocity) than for helices (≈65%) in cell culture media (M2 and TCMair). Apparently, the additional drag that arises from the captured cargo is less of a hindrance for the spirals, as they enclose the cargo and transport it close to their center of rotation by the described rolling motion, whereas helices must push it in front of them, subjecting the entire front surface of the cargo to drag, which significantly increases the microcarrier's front face (from a ring to a disc‐shaped cross section). In addition to the velocity decrease of both micromotors when swimming in a high‐viscosity medium (documented in Figure 3), high viscosity also influences the captured cargo and thus further reduces the velocities of the loaded micromotors, as indicated by the blue columns in Figure 4C, indicating ≈65% for spirals and ≈50% for helices compared with their respective velocities before coupling in a high‐viscosity medium. The relative decrease compared to the velocities after coupling in cell culture medium was similar for spirals and helices, that is, the ratio between pink and blue columns, for spiral micromotors (≈1.4) was comparable to that for helical micromotors (≈1.3). That is, spirals perform better with cargo than helices in general, yet the cargo transport capability of both micromotors is restricted in a similar proportion when the viscosity of the fluid in which they move increases. These observations are supported by ANSYS simulations, as indicated in **Figure** **5** for loaded and unloaded spirals and helices in water‐based medium (*η* ≈ 1 mPa s). The images in Figure 5A (spirals) and Figure 5B (helices) display streamlines indicating fluid velocity distributions after five complete rotations of the respective micromotor rotating at 1 Hz, a rotation frequency that both types of micromotors could follow accurately in MCM. In both cases, the cargo creates additional hydrodynamic flow resistance, as the isotropic shape of the spherical load cannot contribute any potentially beneficial drag anisotropy. However, in the spiral, the coupled cargo does not cause significant disturbances of flow velocity streams in and around the micromotor (Figure 5A), presumably because it aligns well with the shape of the rotating carrier. Conversely, when a helix is pushing the cargo, the streaming route of the flow through the helix lumen, which provides a weak vacuum effect that fixes the cargo at the helix opening as long as it continues rotating, becomes primarily blocked by the cargo particle or cell, and fluid is pushed to the sides before realigning behind the cargo, resulting in a disturbance and loss of kinetic energy (Figure 5B, marked with an arrow). Consequently, the modeled velocities with and without cargo reflect the significant difference already presented experimentally in Figure 4C, whereas spirals do not exhibit such an effect as their motion is dominated by the previously described stepping propulsion. Although the streaming of the fluid flow within the helix lumen aids in holding a pushed cargo particle in place, it is not sufficient to make this coupling resilient and reliable, and it hinders the helix's propulsion significantly, unlike the cargo coupling in the spiral micromotor case.

### 2.4 Cargo‐Loaded Spiral Transfer between Different Environments

**Figure 6:**
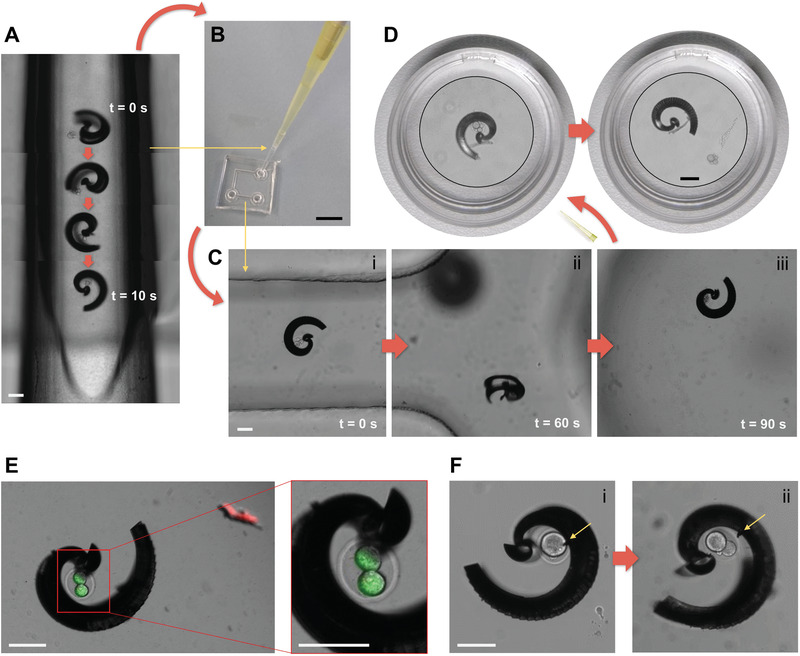
Transfer of cargo‐loaded spiral between different environments: A) Capture of murine zygote inside of trimmed 10 µL pipette tip; B) transfer of cargo‐loaded spiral to PDMS channel by pipetting; C) transport of zygote to other end of channel by magnetic actuation (i–iii); D) transfer of cargo‐loaded spiral to Petri dish by pipetting and release of zygote by magnetic actuation; E) fluorescence staining indicating viability of one cleaved zygote after capture (time taken: 4 min), magnetic manipulation (time taken: 8 min), and 24 h of incubation while inside the spiral in PDMS channel; F) successful cell division of one zygote, depicting the zygote after capture (time taken: 3 min) and magnetic manipulation for 3 min (i), and after 24 h of incubation while inside the spiral (ii) in PDMS channel (yellow arrows mark a piece of debris of spiral's metal coating that indicate that indeed the same spiral is depicted); all scale bars are set at 100 µm except in (B) 1 mm, and in (D) Petri dish diameter is 3 cm—spiral and zygote are displayed in magnified insets.

Spirals perform better with cargo than helices in terms of micromotor velocity, yet more importantly, because they can enclose the cargo and do not lose it unintentionally during magnetically actuated transport. This ability is especially important considering the intended application of micromotor‐assisted ZIFT, where achieving controllable and safe zygote transport in the fallopian tube and protecting the zygote and cell‐to‐micromotor coupling while a loaded spiral is injected into the uterus is critical. This was simulated by transferring cargo‐loaded spirals between different environments by pipetting. In a proof‐of‐concept experiment, a murine zygote was captured by magnetically actuated rotation of a spiral inside a 10 µL pipette tip under the microscope (**Figure** **6**A; Video S9, Supporting Information). The pipette tip was then attached to a larger tip (100 µL) to pipette the loaded spiral into a PDMS channel, together with cell culture medium (Figure 6B). In Figure 6C‐i–iii, the transport of the captured zygote by the magnetic spiral through the PDMS channel is indicated in three panels, covering a distance of ≈2.5 cm in 90 s (Video S9, Supporting Information). Another transfer was performed by pipetting the loaded spiral directly from the channel outlet to a Petri dish with cell culture medium. Figure 6D displays the cargo coupling intact, and the spiral and zygote after magnetically actuated cargo release. Similar experiments were performed with PS particles in MCM to further validate the capability of spiral‐shaped micromotors to enclose and protect cargo even during transfer by pipetting. A helical micromotor can always push cargo; however, it immediately loses contact with the cargo once it stops moving. Conversely, although a spiral must properly capture a cargo first, it provides a reliable and secure coupling that can withstand fluid flow and mechanical impacts. In that respect, the size adaption of spirals proved to be critical. Although larger spirals (tubular diameter 170 µm) that were meant for the transport of PS particles and bovine oocytes/zygotes could also capture murine oocytes and zygotes, the cells were lost during pipetting as they slipped out of the spirals in the process. Conversely, although smaller spirals (tubular diameter 110 µm) could capture murine oocytes/zygotes, the cells could not be released after transport and transfer by magnetically actuated (counter) rotation; that is, the cells remained stuck in the spirals. Spirals with a tubular diameter of 130 µm proved to be most suitable for the reversible capture and release of murine oocytes and zygotes, providing an opening diameter sufficiently large to enable quick and reliable cargo capture and release (on the order of minutes), and sufficiently tight to secure the cargo inside the cavity on demand. The presented resilient and reversible capture, transport, and transfer of cellular cargo by size‐adapted spiral‐shaped micromotors serves as a proof‐of‐concept of micromotor‐assisted ZIFT. The abovementioned performance in high‐viscosity medium and confined tubular channels is the first step toward the in vivo applicability of that concept. An important prerequisite of the proposed application is biocompatibility, especially regarding the captured zygote, which must not be influenced in any detrimental manner by the moving micromotor. For the micromotors presented in this work, only materials that have been reported to be nontoxic and biocompatible elsewhere (Ormocomp resist^[^ ^55^ ^]^ and metal coatings of titanium or tantalum, and iron^[^ ^56^ ^]^) were used. Concerning the viability of zygotes during magnetic manipulation, a detailed analysis of the influence of the micromotor's rotational motion on subsequent embryo development of the manipulated zygote is necessary. At this time, two instances of intact zygote viability after magnetic manipulation are displayed in Figure 6E,F. Figure 6E depicts a cleaved zygote (2‐cell embryo) that was captured by a spiral‐shaped micromotor, magnetically rotated and propelled for 8 min, and then incubated for 24 h (while remaining in the spiral cavity) before a green/red (live/dead) fluorescence staining was applied (please refer to the Experimental Section for more details). The cell assumed the green staining, that is, remained viable, whereas the red staining only accumulated on debris in the microchannel. Figure 6F‐i depicts another zygote that was captured and incubated as in the aforementioned case; however, this was performed one day earlier, that is, before cell division. The zygote divided during incubation for 24 h while remaining in the spiral cavity, as indicated in Figure 6F‐ii. The yellow arrows mark a small piece of debris from the spiral metal coating that remained present during the entire experiment. These two examples present the first indication that zygotes are not harmed by the rotational motion and manipulation executed by spiral micromotors. Video S10, Supporting Information, also displays excerpts of the described magnetic manipulation in these cases (in the video, Example 1 corresponds to the case depicted in Figure 6F and Example 2 corresponds to Figure 6E) in in vitro cell culture medium. By estimating the actual micromotor velocity in real high‐viscosity oviduct fluid to be approximately 100 µm s^−1^, a distance of 4.8 cm could be covered in the described 8 min of magnetic manipulation, which would be sufficient to traverse the uterotubal junction and isthmus length of a human fallopian tube.^[^ ^25^, ^54^ ^]^ The proposed mechanism of cargo capture and release by spirals not only proved its advantage over the established helical micropropeller geometry, but also represents a promising approach for cargo transport by micromotors for biomedical applications in general (for example, targeted delivery of therapeutics or stem cells). It is simple and reliable, easy to control, and only dependent on a rotating magnetic field, with no other physical or chemical triggers or complicated material compositions and arrangements necessary. Rotating, homogeneous magnetic fields are preferable to gradient fields that simply pull a microcarrier, as considerably lower magnetic flux densities are sufficient to penetrate tissue and actuate the micromotor.^[^ ^57^ ^]^ This actuation and the choice of materials ensure the biocompatibility of the system, underlining its potential for in vivo application in high‐viscosity body fluids. To realize this potential regarding the described functionality and controllability, a means of live, high‐resolution, deep‐tissue in vivo imaging and tracking of the applied micromotor is a prerequisite for future in vivo experiments, for example, animal testing. At this time, we must refer to ongoing research in this field, for example, on techniques such as photoacoustic imaging,^[^ ^58^ ^]^ noting that the micromotors presented in this work are in the resolution range of this state‐of‐the‐art in vivo imaging modality and thus could be tracked with this technique in future studies.

## 3 Conclusion

In this study, we propose a novel magnetic micromotor that can capture and transport fertilized oocytes individually in a reversible, controllable, and resilient manner, actuated solely by a homogenous, rotating magnetic field. The propulsion and cargo transport capability were demonstrated in a high‐viscosity medium and confined microfluidic channels, and the safe transfer of cell‐loaded micromotors between different environments was demonstrated for in vivo application for noninvasive, micromotor‐assisted ZIFT. The advantages of the innovative spiral design over the established helical micropropeller geometry regarding cell capture, transport, and transfer performance were verified experimentally and with fluid dynamics simulations. Compared with other strategies of cargo transport by micromotors, the demonstrated approach does not suffer from the requirement for an external stimulus such as light, temperature, a certain chemical environment, or a fragile arrangement of components, which can be difficult to implement in vivo; rather, it can easily capture, transport, and release cellular cargo by magnetically induced rotation. In the future, regarding the in vivo application of the presented micromotor‐assisted ZIFT concept, we may work on the implementation of an in vivo imaging modality, based on the recent work done in the field^[^ ^58^ ^]^ and the utilization of a biodegradable composite material to avoid possible issues with micromotor retrieval after successful zygote delivery. The advantage of in vivo embryo development after noninvasive, micromotor‐assisted ZIFT toward higher success rates of assisted reproductive technology can then be asserted.

## 4 Experimental Section

##### Fabrication of Microfluidic Channels

Microfluidic channel platforms to investigate micromotors and cells under the microscope were fabricated from PDMS on glass or parafilm (Parafilm, Merck KGaA, Germany) between two glass slides. Base and curing agents for the PDMS were poured into a custom‐made poly(methyl methacrylate) (PMMA) mold and cured for 12 h at 65 °C. The obtained channels were then cut to fit onto glass cover slips and pierced with a 2 mm punch to obtain inlets and outlets before fixing them on the glass. Tight fixation was achieved by subjecting both surfaces, channel and glass, to O_2_ plasma (Femto system, Diener Electronic GmbH & Co. KG, Germany) for 30 s before pressing them together manually and curing the bonding for 1 h at 65 °C. Parafilm channels were fabricated by folding a strip of parafilm into three layers and cutting the desired channels into it with an electronic cutting machine (Silhouette CAMEO, Silhouette America Inc., USA). The stacks of layers with the channel shapes were then pressed and fixed between a glass slide and glass cover slip by partial melting and bonding for 2 min at 120 °C on a hot plate. Commercially available (VWR International GmbH, Germany) polypropylene 10 µL pipette tips and elastic PTFE tubing (inner diameter, 500 µm) were utilized as the tubular microfluidic channels for the micromotors by trimming them to a length of ≈2 cm and fixing them onto glass cover slips with adhesive tape. All channels were filled with pluronic (Pluronic F‐127, Merck KGaA, Germany) solution (10 µg mL^−1^ in deionized water) and incubated overnight at 37 °C, then rinsed with deionized water and sterilized under UV light before use. Examples of the different channels are depicted in Figure S2, Supporting Information .

##### Fabrication of Micromotors

Helices and spiral‐shaped micromotors were designed and programed in a general writing language editor (DeScribe, Nanoscribe GmbH, Germany) for direct laser writing (DLW), a 3D laser lithography technology based on two‐photon absorption and polymerization of negative tone photoresist (Photonic Professional GT 3D, Nanoscribe GmbH, Germany). Ormocomp (Micro Resist Technology GmbH, Germany) was employed as a photoresist, drop‐cast onto fused silica substrates, and developed for 10 min (mr‐Dev 600, Micro Resist Technology GmbH, Germany) after DLW‐patterning according to the programed scripts. The developed samples were immersed in isopropanol and dried in a critical point drying machine (EM CPD300, Leica Microsystems GmbH, Germany). Then, the helices were coated with 10 nm Ti, 100 nm Fe, and 15 nm Ti by electron beam evaporation (PLASSYS Bestek Ltd., France) and the spirals were coated with 10 nm Ta, 100 nm Fe, and 10 nm Ta by sputtering deposition (DCA Instruments Oy, Finland).

##### IVF and Cell Culture

Bovine ovaries were obtained from a local slaughterhouse (Südost Fleisch GmbH, Germany) and oocytes were isolated, cultured, matured, and fertilized by IVF with bovine sperm from a local cattle breeding company (Masterrind GmbH, Germany), following established protocols.^[^ ^59^, ^60^, ^61^ ^]^ A similar procedure was performed with sperm and oocytes from laboratory mice that were sacrificed in the Transgenic Core Facility of the Max Planck Institute of Molecular Cell Biology and Genetics in Dresden by Ronald Naumann according to established protocols.^[^ ^62^, ^63^, ^64^ ^]^ Murine sperm and oocytes were collected from unused sources from running projects of generating mutant and rederiving mouse lines by IVF with frozen sperm in the facility. The Transgenic Core Facility holds active permissions for the work with mouse embryos and works under the principles of the 3Rs^[^ ^65^ ^]^ with animals living under specific pathogen free (SPF) conditions. Before and after the micromotor experiments, the fertilized oocytes were incubated at 39 °C (bovine cells) or 37 °C (murine cells) and 5% CO_2_ in the respective cell culture medium. Fluorescence staining of the cells after the micromotor experiments was applied to indicate cell viability after magnetic manipulation, by directly adding 0.5 µL of a fluorescein diacetate solution (5 mg mL^−1^ in acetone) as green (“live”) and 1 µL of propidium iodide solution (stock from Merck KGaA, Germany) as red (“dead”) staining to the microfluidic channel platform where the micromotor and cell had incubated together for 24 h after magnetic manipulation.

##### Magnetic Actuation

The rotating magnetic field for the actuation of the helices and spiral‐shaped micromotors was generated using a commercial “MiniMag” electromagnetic coil setup (MFG‐100‐i, Magnebotix AG, Switzerland), which was mounted onto an inverted microscope (Eclipse Ti2, Nikon Corp., Japan) to actuate and control the micromotors inside the previously described microfluidic channels under live observation and recording at 10× magnification and ten frames per second (DS‐Qi2 camera, Nikon Corp. Japan). The micromotors were separated from their fused silica substrate after fabrication by gentle swiping with a 10 µL pipette tip after a drop of liquid medium was placed on the respective array of micromotors on the substrate. The following liquid media were used for different experiments: bovine oocyte/zygote cell culture medium (TCMair), murine oocyte/zygote cell culture medium (M2), cell culture medium with 0.6 w/v methyl cellulose (MCM), and real oviduct fluid (OVF), (squeezed from fresh bovine oviducts obtained from Südost Fleisch GmbH, Germany, centrifuged to remove tissue and cell debris). The TCMair and M2 were prepared according to established protocols (see IVF and Cell Culture Section). The micromotor samples were subjected to O_2_ plasma (Femto system, Diener Electronic GmbH & Co. KG, Germany) for 30 s to improve wetting before being suspended in the medium. The separated and suspended micromotors were transferred to the parafilm or PDMS channels or sucked into the PTFE tubing and 10 µL pipette tips serving as the tubular microchannels by pipetting. The PS particles (dark red, 100 µm diameter, Merck KGaA, Germany) and murine or bovine oocytes/zygotes were also added by pipetting with the TCMair or M2 medium, respectively. The micromotors were actuated with magnetic flux densities of 1–20 mT and field rotation frequencies of 0.5–70 Hz, and steered by tilting the axis of rotation of the magnetic field with a graphical user interface (Daedalus, Magnebotix AG, Switzerland) and 3D mouse (SpaceMouse, 3Dconnexion GmbH, Germany) connected to the MiniMag setup. An annotated depiction of the MiniMag setup can be found in Figure S2, Supporting Information.

##### Simulations

Maxwell and fluidic simulations were performed using the ANSYS 17.2 Academic software. For the Maxwell simulations, the microstructure models considered a metal coating of 100 nm Fe with a maximum possible saturation of 2 T, considering the distribution of the magnetic layer on the respective micromotor surface. Torques were extracted with the torque parameter placed in the rotational center of the respective object. The investigated geometries were placed within a boundary box with an applied tangential field of 15 954 A m^−1^ corresponding to the 20 mT maximum magnetic field that was applied by the experimental setup. For the fluidic simulations, the liquid medium parameters were set as time‐transient and pressure‐based. Viscous standard k‐epsilon was selected as the model setting, and scalable wall functions were selected as the near‐wall treatment. Mesh motion was selected as the inner cell zone condition and rotation speeds were parametrically applied over the range of 1–100 Hz. Fluid viscosities were selected as 1 mPa s (water) and 20 mPa s (high‐viscosity medium). Calculations were run over a time of 2 s to stabilize the flow in the model, with time steps of 0.01 s. The mesh size for the fluid simulations was limited to maintain the skewness of the mesh below a factor of 0.8.

##### Statistics

Videos and images of the micromotor experiments were analyzed with Fiji software,^[^ ^66^ ^]^ and micromotor velocities were measured with the MTrackJ plugin by Erik Meijering (https://imagescience.org/meijering/software/mtrackj/). Each individual helix or spiral‐shaped micromotor was analyzed at different actuation frequencies, providing multiple tracks with multiple track points, and therefore average velocities with standard deviations for multiple frequencies. The respective maximum velocities of several helices, (*n* = 14) and spirals (*n* = 9), obtained at different frequencies, are averaged (with standard deviation) and summarized in Figure 3A. Cases of helices (*n* = 11) and spirals (*n* = 4) where the cargo was transported by the same micromotor and distinctive tracks before and after cargo coupling were successfully recorded are summarized analogously in Figure 4C, normalized by setting all individual velocities before coupling to one. The standard deviations displayed in both graphs permit the obtained conclusions.

## Conflict of Interest

The authors declare no conflict of interest.

## Supporting information

Supporting Information

Supplemental Video 1

Supplemental Video 2

Supplemental Video 3

Supplemental Video 4

Supplemental Video 5

Supplemental Video 6

Supplemental Video 7

Supplemental Video 8

Supplemental Video 9

Supplemental Video 10
