# Supplementary material for: A Rotating Spiral Micromotor for Noninvasive Zygote Transfer
Source: Adv Sci (Weinh). 2020 Jul 21;7(18):2000843. doi: 10.1002/advs.202000843 (PMC7509649; doi:10.1002/advs.202000843)
Supplement: Supplementary file 1 — Supporting Information [file ADVS-7-2000843-s001.pdf]

Copyright WILEY-VCH Verlag GmbH & Co. KGaA, 69469 Weinheim, Germany, 2020.

Supporting Information

A Rotating Spiral Micromotor for Noninvasive Zygote Transfer

*Lukas Schwarz, Dmitriy D. Karnaushenko, Franziska Hebenstreit, Ronald Naumann, Oliver G. Schmidt, and Mariana Medina-Sánchez**

Micromotor designs and dimensions

The dimensions of the helical micromotors as they were fabricated by direct laser writing (DLW) are given in **Figure S1 A**. The spiral-shaped micromotor dimensions are listed in **Figure S1 B**, indicating four different sizes that were scaled proportionally to capture different types of cargo. The micromotor wall thicknesses thereby remained constant. The curved walls and helical fin structure along the backbone of a spiral were built from blocks of lines that were exposed, one at a time, by the pulsed laser for cross-linking, resulting in a certain roughness of the structure, especially regarding the fin. The fin width also remained constant for all four spiral sizes, with its maximum width at the spiral opening, decreasing from there to 0 µm close to the spiral's origin, that is, center of rotation. The helix and spiral shapes as they were programed by DeScribe (Nanoscribe GmbH, Germany) for DLW are displayed in **Figure S1 C and D**, respectively. Please note that both microstructures were fabricated being attached to a fused silica substrate and were later separated from that substrate by gentle swiping with a polypropylene (PP) pipette tip. This swiping procedure typically cuts off the bottom ring on which the helices stand (Figure S1 C) such that the separated helices feature only one remaining ring at their top, as intended. The spirals typically remained undamaged after swiping. Both microstructures were coated with metal multilayers before being separated from their respective substrate. **Figure S1 E** displays the estimated distribution of the metal on the two different micromotor types after coating. Helices were coated by electron beam evaporation and the

samples were tilted by 15° and rotated at 5 rpm during coating, which led to a homogenous distribution of metal over the helix surfaces. The spirals were coated by sputtering deposition, leading to an inhomogeneous distribution of metal due to a shadowing effect of the fin structure along the spiral backbone, as indicated in Figure S1 E. The different coating distributions could be verified by optical microscopy as coated micromotor parts appeared black whereas uncoated parts revealed the transparent photoresist. In **Figure S1 F**, the different coating compositions for the helices and spirals are listed from inner to outer layer.

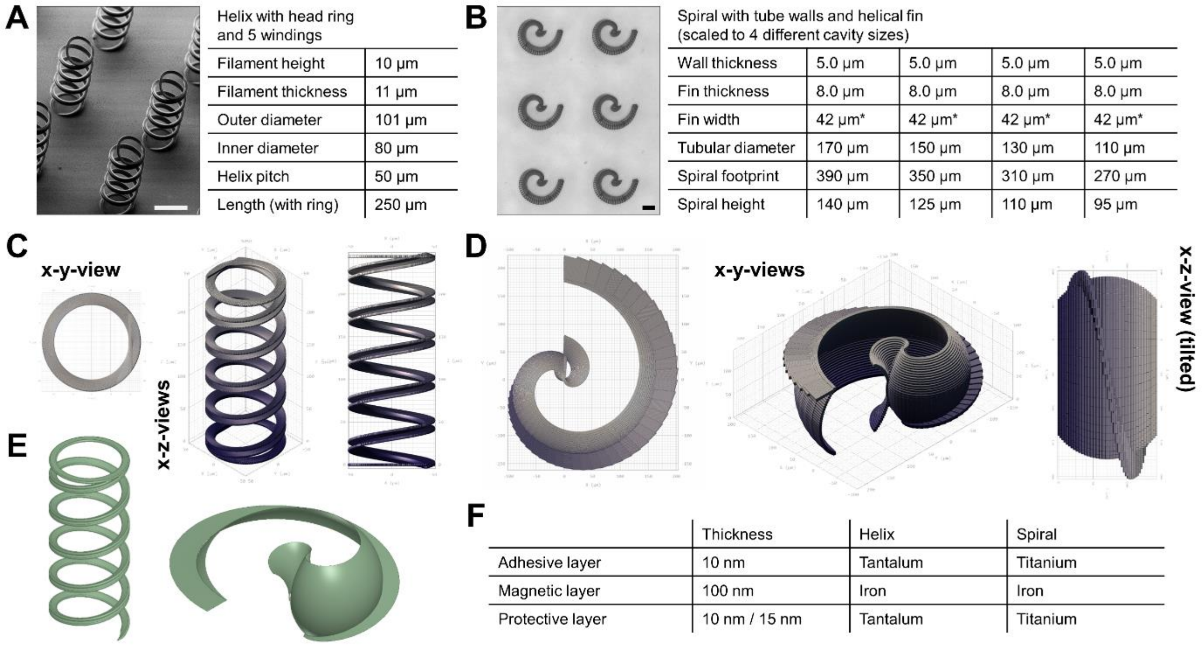

Figure S1. Micromotor designs and dimensions: A) electron microscopy image of helices with given dimensions; B) optical microscopy image of spirals with given dimensions as well as dimensions of downscaled spirals with constant wall thicknesses and fin dimensions (*fin width decreases from its maximum of 42 µm at spiral opening to 0 µm close to spiral’s origin, that is, center of rotation), scale bars 100 µm; C) images of programed helix for DLW; D) images of programed spiral for DLW; E) estimation of coverage of respective micromotor surfaces with magnetic material by electron beam evaporation (helix) and sputtering deposition (spiral) used

in Maxwell simulations (ANSYS 17.2 Academic); F) compositions and thicknesses of metallic multilayers deposited on helices and spirals (listed from inner to outer layer).

Propulsion performance of helices and spirals

The commercial "MiniMag" electromagnetic coil setup that was used with an optical microscope for live actuation and observation of the magnetic micromotors is displayed in **Figure S2 A** with its components listed. The micromotors were operated in a liquid media in microfluidic channel platforms that were inserted into a custom-made sample holder frame that could be mounted onto the microscope stage. **Figure S2 B** displays the design for cutting out the channels from parafilm, and a photograph of a fabricated parafilm channel that is filled with cell culture medium. **Figure S2 C** displays the design for micromachining the poly(methyl methacrylate) (PMMA) mold that was used for the fabrication of the polydimethylsiloxane (PDMS) channels, and a photograph of a fabricated PDMS channel with punched inlets/outlets. In **Figure S2 D and E**, photographs of trimmed polytetrafluorethylene (PTFE) tubing and a trimmed PP 10 µl pipette tip are displayed, respectively. These were used for the investigation of the micromotor propulsion under spatial confinement. Whereas the parafilm and PDMS channels featured widths and heights of 1 mm (and greater), the PTFE tubing and PP pipette tips embodied tubular channels with cross section diameters down to 500 µm.

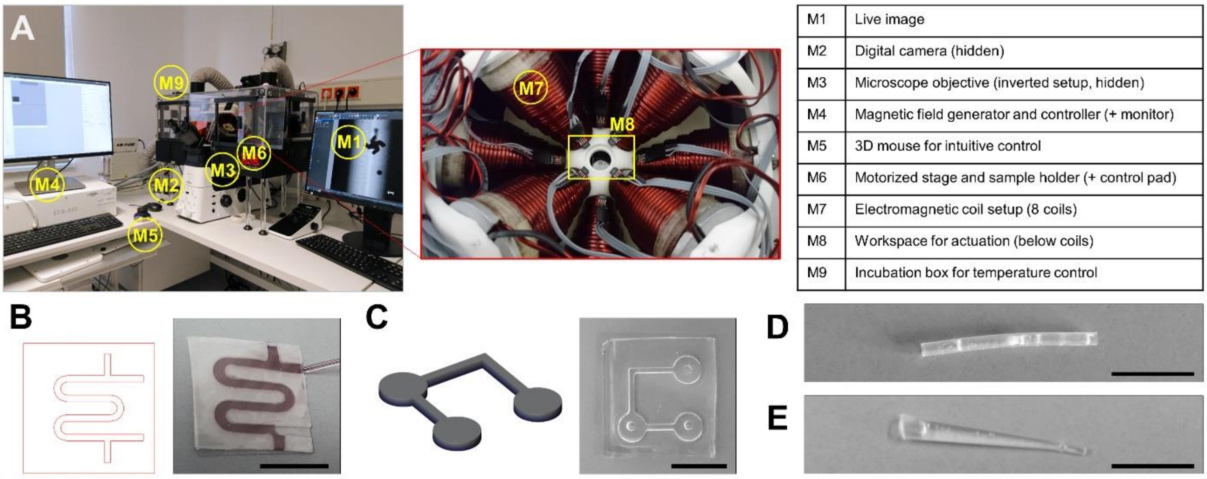

Figure S2. Magnetic actuation and observation of micromotors: A) "MiniMag" setup with magnetic field generator and optical microscope for live actuation, observation, and video recording of micromotors; B) design and implementation of parafilm channels for experiments with helices; C) design and implementation of PDMS channels for experiments with spirals; D) trimmed PTFE tubing to investigate micromotor propulsion in confined channels; E) trimmed PP 10 µl pipette tip to investigate influence of varying confinement on micromotor propulsion; all scale bars 1 cm.

The details of the propulsion of the spiral-shaped micromotors are illustrated in a series of videos. Please note that all videos (except simulations) were recorded with inverse microscopy, that is, from below the respective microfluidic channel platform.

- **Video S1** indicates the in-plane rotation and upright rotation of a spiral in water at 4 Hz, recorded at 30 fps. Only the latter case exhibits efficient forward motion.
- **Video S2** displays simulations by the ANSYS 17.2 Academic software of the fluid flows around a spiral when rotating in-plane and upright. The in-plane rotation is viewed from the top of a channel with rectangular cross section of 1 mm width and height; upright rotation is viewed from the side of a similar channel.
- **Video S3** indicates the tracking with the MTrackJ plugin in Fiji of a spiral moving in water at 1 Hz, recorded at 30 fps. A track mark was placed every 15th frame, that is, after each half-rotation of the spiral, to illustrate the uneven hobbling motion of the spiral.
- **Video S4** displays four examples of spiral and helix propulsion in different environments, recorded at 10 fps. In each example, the respective micromotor was operated to achieve its maximum possible velocity.
- **Video S5** displays two examples of one and the same spiral swimming in the bulk of the fluid (water), not in contact with the channel floor, at 30 Hz, recorded at 30 fps. The

spiral rotates evenly, gliding smoothly through the fluid, albeit not particularly fast despite the relatively high frequency. Please note that because of the relative synchronicity of rotation frequency and video frame rate, the spiral appears to be rotating considerably more slowly than it actually did. In fact, if it had been able to accurately follow the externally applied magnetic field rotation of exactly 30 Hz, it would not appear to be rotating at all in a video at 30 fps.

- **Video S6** is two examples of a spiral moving back and forth, both at 5 Hz, recorded at 30 fps. In both examples, the spiral is first steered smoothly to make a U-turn and then forced to backtrack by instantly switching the direction of rotation of the externally applied magnetic field, both recorded at 30 fps. In the first example, a spiral moving in water falls into a tumbling mode when the direction of the rotation of the magnetic field is suddenly switched and then flips its orientation to realign to the new direction of rotation, moving in the same fashion as it did before the switch, yet in the opposite direction. In the second example, a spiral moving in MCM does not go into tumbling and reorientation after the magnetic field rotation is switched; it simply inverses its rotation. However, this does not lead to efficient forward propulsion as the crucial "hobbling step" component of spiral propulsion is absent in this configuration.

Further details on the propulsion of both helices and spirals are exemplified in **Figure S3**. **Figure S3 A and B** illustrate the relation between actuation frequency, that is, rotation frequency of the externally applied magnetic field, and micromotor velocity of one helix in water and in methyl cellulose-containing medium (MCM) with ca. twentyfold viscosity, respectively. The blue and green data points reflect experimentally obtained values; red data points represent data from the ANSYS simulations. Both Figure S3 A and B indicate the known linear increase of velocity with frequency up to a commonly called step-out frequency that is characteristic for helical micromotors[23,40,44] and is highlighted yellow in the respective graphs. The modeled data in red serves well to reflect that linear increase. However, it does not provide

a correct estimation of the step-out frequency beyond which the helix can no longer follow the magnetic field rotation and thus does not propel efficiently, as the experimental data indicates. In Figure S3 B, two distinct series of experimental data of one and the same helix are displayed, one that corresponds to swimming, that is, corkscrew propulsion (as in Figure S3 A), and one that reflects rolling, that is, when the helix was moving close to the substrate floor with significant side drift, rolling sideways as a cylinder rather than propelling forward as a corkscrew. Rolling is faster than swimming; however, it is more difficult to control experimentally in the case of the helix. **Figure S3 C and D** depict analogous frequency-velocity-graphs of one spiral in water and MCM, respectively. The spiral-shaped micromotors are propelled forward with the ascribed “hobbling step” motion that can be roughly understood as rolling. Consequently, Figure S3 C reflects a linear increase of velocity with frequency, as observed with the propelling as well as rolling helices, and also described in the literature for other rolling microstructures. However, as reported in the literature,[50,51] there is no distinct step-out frequency; rather a plateau regime after a certain frequency (again highlighted yellow) where the micromotor velocity remains approximately constant for a wide range of higher frequencies. Again, this transition could not be modeled correctly with our simulations, whereas the linear increase regime is reflected rather accurately. In a high-viscosity fluid, a linear regime could not be observed experimentally at all, as the plateau regime already started at 1 Hz, the lowest applied frequency, in MCM (Figure S3 D). Therefore, the data obtained by the simulations was not suited to describe these results, albeit the first data point at 1 Hz is in the range of the experimentally obtained values. The two different series of experimental data in Figure S3 C and D correspond to the motion of one and the same spiral in different regions of the microfluidic channel platform, respectively. The PDMS channels as depicted in Figure S2 C were employed; “channel” describes the interconnecting parts between the inlets with a rectangular cross section of 1 mm, whereas “inlet basin” means the rather unconfined area underneath one of the channel inlets, that is, cylindrical basins with 1 mm height and 5 mm

diameter (see Figure S2 C). Apparently, spiral-shaped micromotors are typically faster in the basins than in the channels, that is, in a more confined environment. This could also be observed experimentally with a spiral that was propelling through a pipette tip (as in Figure S2 E) that served as a tapered tubular channel, that is, with narrowing diameter along the spiral's track. The corresponding data is depicted in **Figure S3 E** and indicates a linear decrease of micromotor velocity with decreasing channel diameter, that is, with increasing confinement. The main factors that determine the end of the linear regimes for both helices and spirals (highlighted yellow in Figure S3) are fluid viscosity, micromotor shape, and magnetization.[42–46,48,49] Spirals achieve greater velocities and different transition frequencies compared to helices because of their different architecture that results in a different translation of rotation to forward propulsion in a rotating magnetic field. For both micromotors, a greater viscosity of the surrounding fluid impedes their motion because of a higher counter-torque from the fluid. This high fluidic drag must be matched with a high torque evoked by the rotating micromotor; however, the maximum torque it can evoke is limited by its maximum magnetization, as it must be able to synchronously follow the torque imposed by the rotating, externally applied magnetic field. **Figure S3 F** displays the maximum possible torques of the rotating helices and spirals, modeled by ANSYS with maximum magnetizations derived from estimations regarding Figure S1 E. Unfortunately, the derived model could not serve to properly limit the linear regimes of the micromotor propulsion, as indicated by the red data lines in Figure S3.

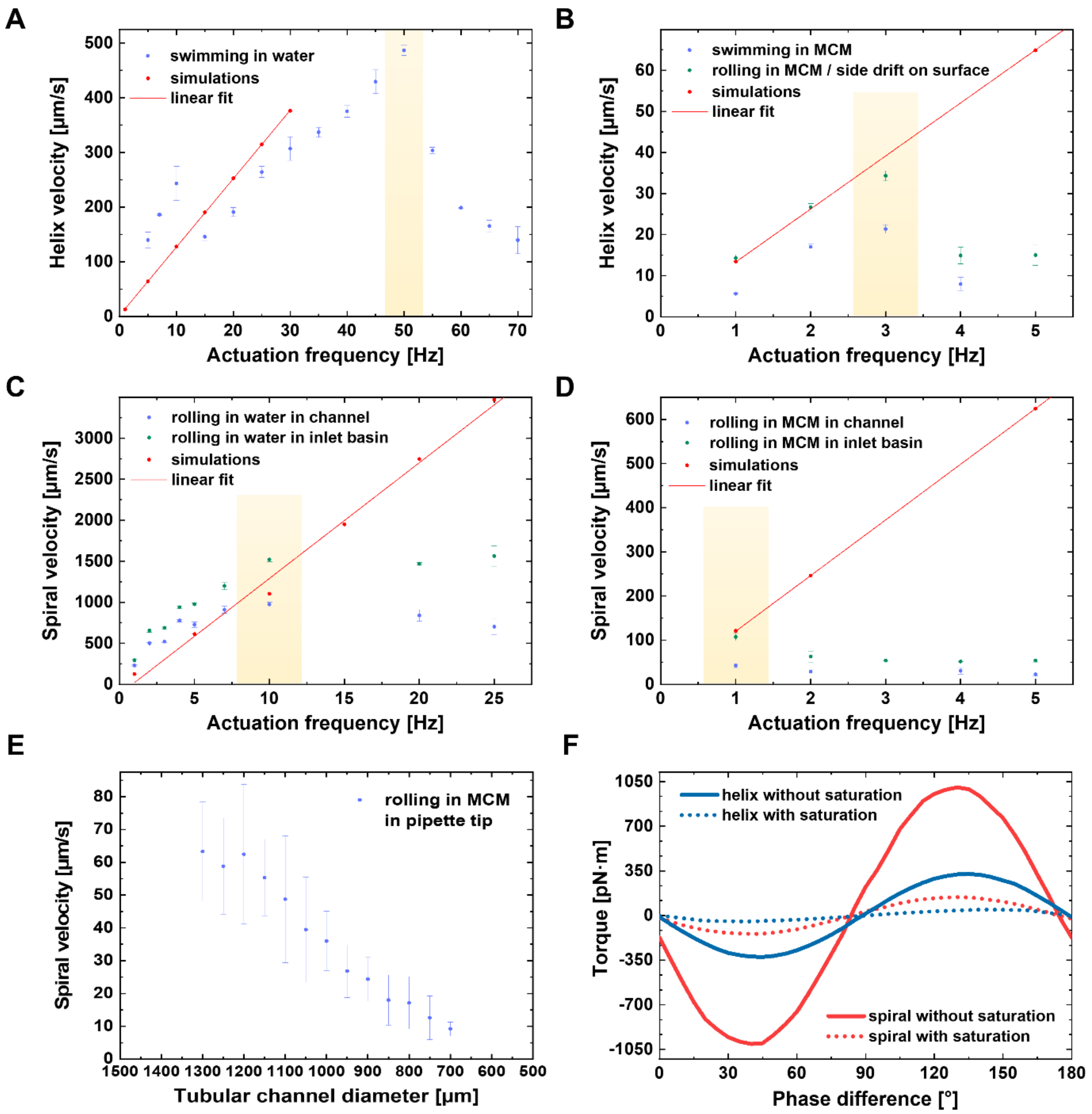

Figure S3. Examples of propulsion performance of helices and spirals: A) helix in water; B) helix in MCM; C) spiral in water; D) spiral in MCM; blue and green data points represent experimental results of one specific microstructure (error bars indicate the standard deviation of the velocity tracking along multiple tracks at given actuation frequency), red data points and lines represent modeling results obtained with ANSYS 17.2 Academic, yellow columns indicate transition frequencies from linear velocity increase regimes to decrease after step-out (in case of helices, A and B) or plateau regime (in case of spirals, C and D); E) experimental results corresponding to spiral-shaped micromotor propelling through 10 µl PP pipette tip (with tapering cross-section diameter) at fixed frequency (4 Hz), error bars indicate the standard

deviation of velocity tracking along multiple tracks; F) modeling results (Maxwell simulations with ANSYS) of maximum torque generated by helix and spiral-shaped micromotors in homogeneous magnetic field of 20 mT with respect to saturation magnetization and resulting phase difference between rotating magnetic field and respective microstructure with estimated distribution of ferromagnetic material as indicated in Figure S1, E.

Cargo transport with helices and spirals

The coupling and transport of the cellular cargo to spiral-shaped micromotors and helices is illustrated in a series of videos. Please note that all videos were recorded with inverse microscopy, that is, from below the respective microfluidic channel platform.

- **Video S7** displays the capture, transport, and release of a murine oocyte with one spiral in M2 medium in a PDMS channel, recorded at 10 fps. The actuation frequency was varied in the range of 0.5–5 Hz in the process of completing these tasks.
- **Video S8** displays three examples of bovine zygotes being transported by individual helices, recorded at 10 fps. The examples serve to illustrate the loose coupling between the cargo and helix that necessitates frequent realignment and recapture during transport, despite a microfluidic vortex established within the helix lumen that aids in holding the cargo in place at times.
- **Video S9** displays the stages of capture, transport, and release of a murine zygote with one spiral that involves the transfer of the cargo-loaded spiral between different environments, recorded at 10 fps. Please note that the magnetically induced release of the zygote after successful transfer to a culture dish could not be recorded live because the culture dish did not fit into the sample holder of the MiniMag setup. The cargo-loaded spiral was transferred to another microfluidic channel platform instead, where the zygote was released by magnetically induced rotation, and then the separated cell and micromotor were transferred back to the culture dish to obtain the final image.

- **Video S10** displays two examples of murine zygotes being captured and manipulated by individual spiral micromotors, recorded at 10 fps. Both zygotes were subjected to live/dead (green/red) fluorescence staining after being captured by the respective spiral and incubated subsequently for 24 h. On Day 1 of the recorded experiment, the first zygote was captured by a spiral in a PDMS channel platform and incubated (Example 1). On Day 2, this zygote divided (while inside the spiral), and was stained and observed again. On Day 2, the second (already divided) zygote was captured in another PDMS channel platform by another spiral, actuated for several minutes (excerpts are indicated in the video), and incubated (Example 2). On Day 3, the zygote was stained and observed again (only a still frame of the fluorescence imaging procedure is displayed in the video).

Fluid dynamics simulations by ANSYS 17.2 Academic software are depicted in **Figure S4 A** for spirals and **Figure S4 B** for helices with and without cargo, respectively. The images illustrate the flow velocities of fluid streams around the rotating micromotors. The high-viscosity medium was modeled with a viscosity of 20 mPa·s, water-based medium with 1.0 mPa·s. In different media, different maximum micromotor rotation frequencies can be achieved; therefore, the results for 1 Hz rotation are indicated in a high-viscosity medium, and 30 Hz rotation in a water-based medium for both types of micromotors, which corresponds to the experimentally obtained results. The simulations indicate greater velocities of both micromotors in the water-based medium compared to the high-viscosity medium, as well as a significant disturbance of the fluid flow streams when the helices are pushing spherical cargo in both media.

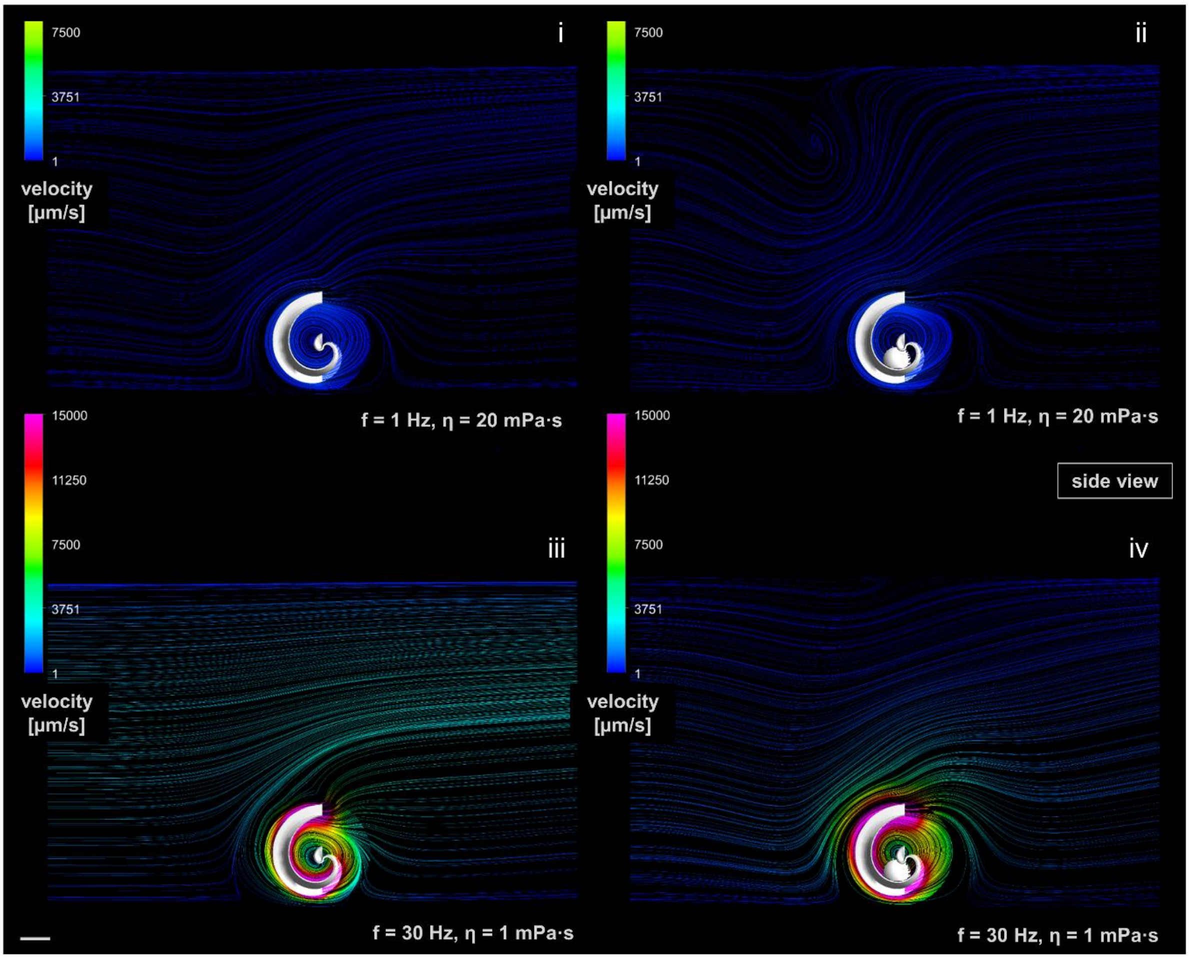

Figure S4 A. Fluid dynamics simulations (ANSYS) of unloaded (i, iii) and loaded (ii, iv) spirals in high-viscosity medium at 1 Hz rotation (i, ii) and water-based medium at 30 Hz rotation (iii, iv) after five complete rotations, all in side view with spirals in channel with rectangular cross-section of 1 mm width and height, respectively; color scales of flow velocities around spirals are logarithmic (top legends are capped and correspond to bottom ones), scale bar 100 µm.

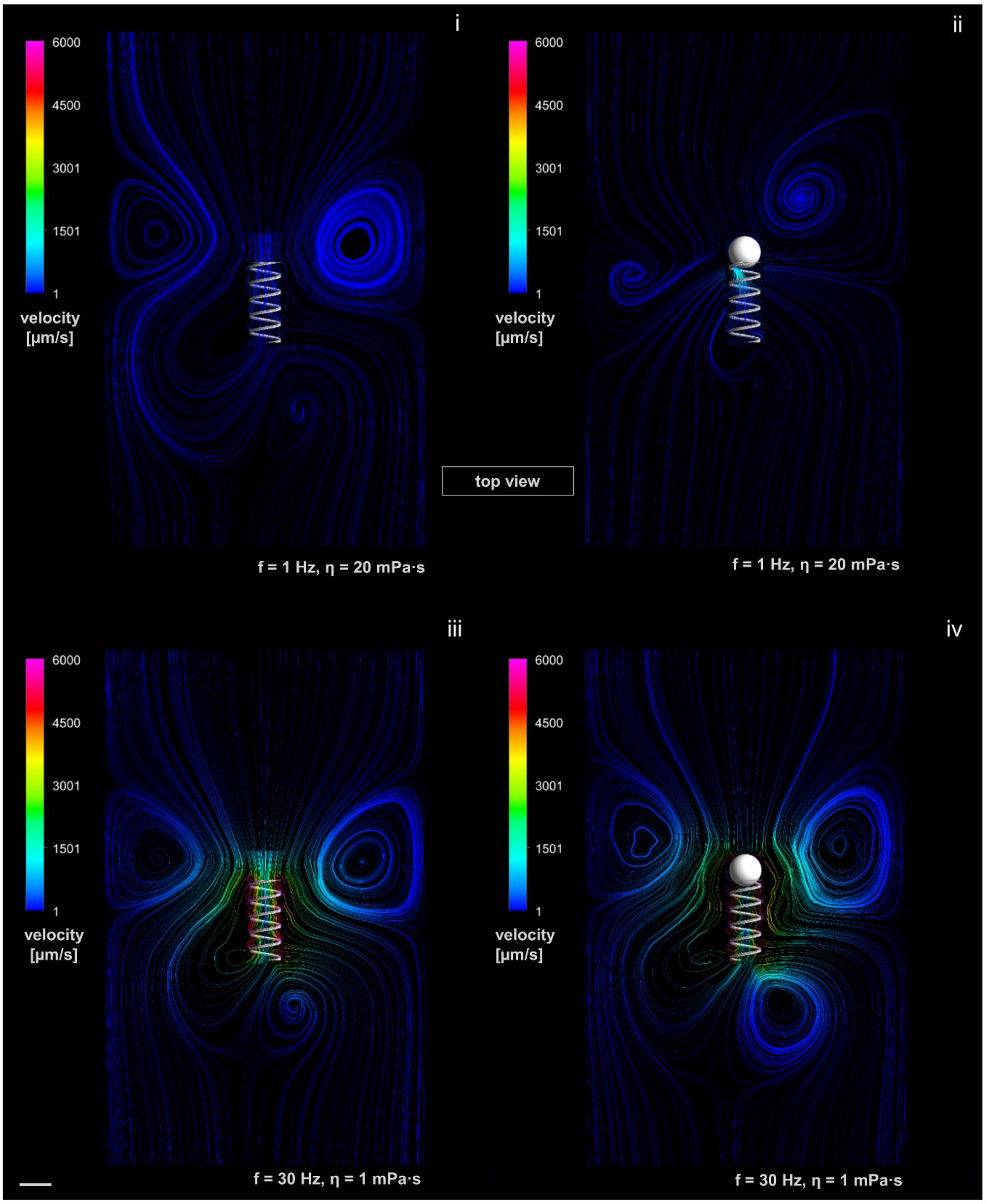

Figure S4 B. Fluid dynamics simulations (ANSYS) of unloaded (i, iii) and loaded (ii, iv) helices in high-viscosity medium at 1 Hz rotation (i, ii) and water-based medium at 30 Hz rotation (iii, iv) after five complete rotations, all in top view with helices in channel with rectangular cross-section of 1 mm width and height, respectively; color scales of flow velocities around helices are logarithmic, scale bar 100 µm.
